# Can we recommend varicocele surgery for men with hypogonadism?

**DOI:** 10.1590/S1677-5538.IBJU.2023.0190

**Published:** 2023-06-20

**Authors:** Nilson Marquardt, Carlos Teodósio Da Ros

**Affiliations:** 1 Pontifícia Universidade Católica do Rio Grande do Sul Departamento de Urologia Porto Alegre RS Brasil Departamento de Urologia, Pontifícia Universidade Católica do Rio Grande do Sul, Porto Alegre, RS, Brasil; 2 Universidade Luterana do Brasil Disciplina de Urologia Canoas RS Brasil Disciplina de Urologia, Universidade Luterana do Brasil – ULBRA, Canoas, RS, Brasil

## COMMENT

Varicocele is a frequent finding, identified in 15% of the male general population and, among those with infertility, the prevalence is around 35% for primary infertility and 80% for secondary infertility (1, 2). This pathology can be defined as an abnormal tortuous dilation of internal spermatic veins and pampiniform plexus in spermatic cord. Some risk factors such as age, height, lifestyle habits are associated with the presence of varicocele, however it seems that an increase of body mass index decreased risk of varicocele (3). The early diagnosis and treatment aim to avoid the possible progressive duration-dependent reduction in testicular function, demonstrated as impaired semen parameters, low testosterone (TT) levels and increased sperm DNA fragmentation levels, particularly stress-induced sperm DNA damage (1, 4, 5).

Many hypotheses have been proposed to explain the consequences of varicocele in testicular function, such as testicular hyperthermia, hypoxia, hormonal dysfunction, decreased blood flow, backflow of metabolites from the adrenal gland, impairment of Leydig cells (6–8). Furthermore, Weiss et al. reported a reduced testosterone synthesis in individuals with varicocele (9). Another study suggests that Leydig cell hyperplasia, commonly seen in patients with varicocele, is a compensatory reaction to Leydig cell dysfunction and impaired testosterone synthesis (10).

In men presenting with both hypogonadism and varicocele, the stimulation of hypothalamic-pituitary-gonadal axis with gonadotropins or clomiphene citrate is feasible. If combined with varicocelectomy, the serum levels of TT can be further improved. However, the real benefit of this treatment remains unclear (11, 12). The treatment of the varicocele will at least stop possible further varicocele-induced testicular damage and, in a majority of men, lead to improved semen parameters (13), enhanced Leydig cell function in association with increased TT levels and as a consequence, improved quality of the semen due to the reduction in DNA damage (10, 14).

The present review compiles the effects of varicocelectomy in men with hypogonadism on TT serum levels.

## MATERIALS AND METHODS

**Figure 1:**
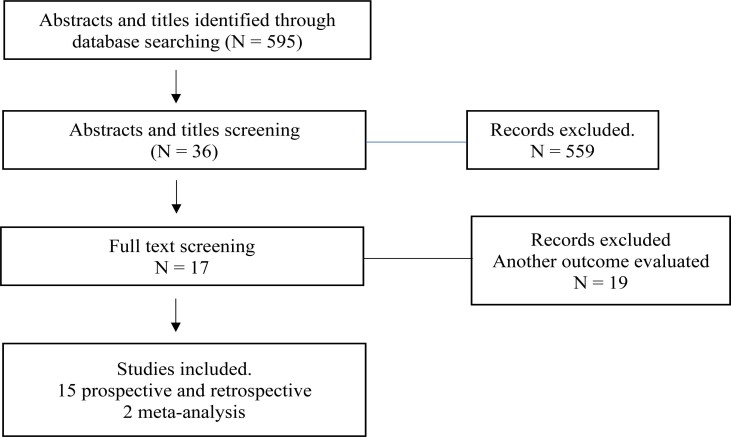
Flow chart of screened and excluded publications.

We selected articles published from 1975 to 2022. Studies were obtained using PubMed. The search terms “varicocele”, “hypogonadism” and “testosterone level” were used as filters. The language of publication was set only to English. In total, 595 potential articles were found. Through screening of titles and abstracts, 36 articles were identified. After full-text reading, 15 original studies and 2 meta-analyses were included in this review (Figure-1).

## RESULTS

Several studies demonstrated that varicocele might be associated with decreased testosterone production and impaired sperm synthesis, corroborating with the hypothesis that varicocele may be linked with hormonal dysfunction. The first study published by Comhaire et al. in 1975 demonstrated that 10 of the 33 men with varicocele analyzed had decreased testosterone levels and erectile dysfunction, and both symptoms were improved in those submitted to surgery (12). In 1978 Rodriguez-Rigau et al. reported that individuals with varicocele and normal serum levels of TT submitted to varicocelectomy showed decreased Leydig cell counts on testicular biopsy (15).

Su et al. reviewed the effect of varicocelectomy on TT levels in 53 men and showed a mean TT level increase from a preoperative level of 319 (±12 ng/dL) to 409 (±23 ng/dL) in the post operatory period (p<0.0004). These findings demonstrated a direct positive correlation of varicocelectomy with spermatogenic function (16).

In 1999, Cayan et al. analyzed the effects of varicocelectomy on FSH and TT levels. They retrospectively evaluated 78 infertile patients who underwent microsurgical inguinal varicocelectomy. The mean FSH levels decreased from 15.21 mIU/mL to 10.82 mIU/mL after surgery, and mean TT levels increased from 563 ng/dL (± 140) to 837 ng/dL (± 220) (17).

Pierik et al. demonstrated the impact of varicocelectomy on serum inhibin B levels, TT levels, FSH and LH levels. Within the 30 men analyzed, there were no significant changes in serum levels of FSH, LH and testosterone levels, but, instead, a significant increase in inhibin B was observed, from 133.9 (± 13.4 pg/mL) to 167.8 (± pg/mL) (18). Similarly, Di Bisceglie et al. performed sclerotherapy in 38 patients with varicocele and compared inhibin B and FSH levels with 40 untreated patients with varicocele. A significant increase (p < 0.01) in serum inhibin B levels and a significant decrease (p < 0.05) in FSH levels were observed at 6 months after treatment. However, there was no significant increase on TT levels (660 ng/dL before procedure and 650 ng/dL after) (19). Likewise, Ozden et al. studied thirty men presenting with varicocele and infertility associated with oligoasthenospermia. All hormone analysis were performed preoperatively and at a 6-month postoperative follow up. There was no statistically significant difference between the mean serum FSH, luteinizing hormone (LH), prolactin and testosterone levels before and after treatment (p > 0.05). However, they showed a significant improvement in sperm concentration, forward progressive motility, and serum inhibin B levels after treatment (p < 0.05) (20). Furthermore, Rodriguez Peña et al. studied the effects of varicocelectomy in hormone levels and seminal parameters in 202 patients. They did not find any difference between pre and post operative on TT levels (preoperative 648 ± 156 ng/dL, post operative 709 ± 232 ng/dL) (21).

Gat et al. performed a study on internal sperm vein embolization in patients with varicocele and reported that mean serum TT concentration rose after embolization by 43%, from 12.07 to 17.22 nmol/L (p < 0.001). Also, the author demonstrated a significant decrease in the mean serum FSH levels, improving Leydig and Sertoli cell functions (22).

Lee et al. evinced that the mean TT levels of 18 men submitted to microsurgical varicocelectomy and vasectomy increased from 348 ng/dL preoperatively to 416 ng/dL postoperatively (23).

In 2011, Zohdy et al. analyzed 141 infertile men with varicocele. They compared the International Index of Erectile Function score (IIEF-5) and total serum testosterone. The patients were divided into two groups according to surgical intervention; 103 individuals were submitted to varicocelectomy and 38 were the control group (assisted reproduction procedures). They showed that the serum TT significantly increased from 379.1 ± 205.8 before varicocelectomy to 450.1 ± 170.2 ng/dL 6 months after and the IIEF-5 increased from 17.1 ± 2.6 in the initial visit to 19.7 ± 1.8 after the surgery. Interestingly, there was a non-significant negative correlation between the mean change in TT and the mean change in IIEF-5 in the control group (24).

Hsiao et al. retrospectively reviewed 272 patients who underwent subinguinal microsurgery varicocelectomy between 1996 and 2009 and compared serum TT levels before and after treatment. Patients who had baseline TT lower than 400 ng/dL had a greater increase in serum levels (309 ± 7.4 to 431 ± 16.2 ng/dL, p 0.001). And this elevation occurred even in individuals over 40 years old. (25).

Tanrikut et al. measured preoperative TT levels in 325 men with palpable varicocele and in 510 men with vasectomy reversal without varicocele who served as a control group. The TT level increased significantly from 358 ± 126 ng/dL to 454 ± 168 ng/dL after varicocelectomy. Within the subgroup of patients with postoperative TT level improvement, the mean increase was 178 ng/dL. Also, they evidenced that varicocele was a risk factor for androgen deficiency and the microsurgical repair increased hormonal levels (26).

Sathya Srini et al. evaluated 200 men diagnosed with clinical varicocele. They were divided into two groups, one submitted to microsurgical varicocelectomy and the other to assisted reproduction procedures. The group that underwent surgery had an increase in mean TT (177 ± 18 ng/dL before varicocelectomy to 301 ± 43 ng/dL after) and in testicular size (+ 1.508 cc) that was associated with mean TT change. Out of the 100 patients of the varicocelectomy group, 78 had postoperative normalization of TT levels, while only 16 of the control group had the same outcome (27).

Abdel-Meguid et al. performed a prospective controlled study in 171 men divided into four groups: varicocele-infertile treatment group, varicocele-infertile control group, varicocele-fertile treatment group and the normal-control group. They compared TT levels at 6 and 12 months. Significantly lower hormonal level was evidenced in men with varicocele compared with normal men. Varicocelectomy increased TT levels in 102.3 ng/dL among hypogonadal men, however showed no improvement in eugonadal men (28).

Najari et al., in 2016, analyzed 34 patients retrospectively who had undergone microsurgical varicocelectomy and had both pre and post-operative Male Sexual Health Questionnaire (MSHQ). Most men in the study had bilateral varicocele and left grade III varicocele. Significant improvement in the total MSHQ score (3.9 ± 8.7, p=0.027), the MSHQ erectile function (1.2 ± 2.3, p=0.007), and the MSHQ ejaculatory function (1.4 ± 3.1, p=0.018) domains were seen. Fifteen (44%) men reported improvement in their erectile function and 18 (53%) noticed better improvement in ejaculatory function. The mean baseline testosterone level in the 20 men who had post-operative levels assessed was 379.4 ± 164.7 ng/dL and post-operatively mean TT level increased to 515.3 ± 231.6 ng/dL (p=0.007) (29).

Ji et al. conduced a prospective comparative study from 2014 to 2015, comparing 130 men who had varicocele and complained of either infertility or scrotal pain, and 130 controls. All participants were further classified based on hypogonadism status using a serum testosterone cut-off value 300 ng/dL. They could see a correlation of grade II and III varicocele with an increased risk of hypogonadism as well as a correlation of impaired sexual function with TT levels (30).

Recently, Saylam et al. retrospectively analyzed 202 infertile men with hypogonadism and varicocele who underwent microsurgical sub-inguinal varicocele repair. Their hormonal and sexual function after surgery were assessed. Mean serum TT levels increased from 255 ± 66 ng/dL to 372 ± 134 ng/dL (p = 0.000), while serum FSH (p=0.198), LH (p=0.207) and prolactin (p=0.345) levels did not significantly change from pre to post-operative period. Also, IIEF-EF score significantly increased from 27.47 ± 2.96 (15–30) to 28.61 ± 2.02 (18–30), after surgery (p=0.000) (31).

Bernie et al. compared men who underwent varicocelectomy versus testosterone therapy in patients in their 5th and 6th decades of life, and those submitted to the procedure improved as much as younger men. For those with TT < 400 ng/dL larger changes in serum testosterone with baseline normal levels were observed. Also, the improvement may not be clinically meaningful and may not be enough to improve symptoms in some men (32).

A meta-analysis done by Chen et al. in 2017, included eight studies and 712 patients who underwent varicocelectomy. The mean TT levels of patient's post-operation improved by 34.3 ng/dL (p < .00001) compared with their pre-operative levels. In a subgroup analysis, TT improvement in hypogonadal men was more significant (improvement of 123 ng/dL, p<.00001) than in eugonadal men, or in the untreated controls. In an analysis of surgery versus untreated controls (three studies included), results showed that mean testosterone levels among hypogonadal men increased by 105.65 ng/dL, favoring varicocelectomy, as the between groups difference was statistically significant (p < .00001). There were insignificant differences in the eugonadal control group (p = 0.36) (33).

Another recent meta-analysis conducted by Russo et al. comprised fifteen studies, nine were retrospective and six observational. They showed that the mean difference of TT levels was statistically significant in men before and after varicocelectomy (mean difference = 106.76 ng/dL; p < 0.0001). Although a high heterogeneity was present among the studies (34).

Below, we summarized on Table 1 all the studies in this review, reporting the effects of varicocelectomy in total serum testosterone level in hypogonadal men.

**Table 1:** Literature studies evinced the correlation between microsurgery varicocelectomy and serum testosterone levels.

Author	Year	Study type	N. of patients	Surgical Approach	Mean pre-op TT (nd/dL)	Mean post-op TT (nd/dL)
**Su et al. (** 16 **)**	1995	O	53	Microsurgical	319 ± 12	419 ± 23
**Cayan et al. (** 17 **)**	1999	R	78	Microsurgical	563 ± 140	837 ± 220
**Pierik et al. (** 18 **)**	2001	O	30	Conventional	460 ± 160	470 ± 190
**Di Bisceglie et al. (** 19 **)**	2007	O	38	Sclerotherapy	660 ± 50	650 ± 50
**Ozden et al. (** 20 **)**	2008	O	30	Conventional	660 ± 130	720 ± 130
**Rodriguez Pena et al. (** 21 **)**	2009	O	202	Conventional	648 ± 156	709 ± 232
**Lee et al. (** 23 **)**	2007	O	18	Microsurgical	360 ± 191	416 ± 358
**Zhody et al. (** 24 **)**	2011	O	141	Microsurgical	379 ± 205.8	450 ± 170.2
**Hsiao et al. (** 25 **)**	2010	R	272	Microsurgical	309 ± 7	431 ± 170
**Tanrikut et al. (** 26 **)**	2011	R	325	Microsurgical	200 ± 7	454 ± 168
**Sathya Srini et al. (** 27 **)**	2011	O	200	Microsurgical	177 ± 18	301 ± 43
**Abdel-Meguid et al. (** 28 **)**	2014	O	28	Microsurgical	233.8 ± 50.7	327.5 ± 53.2
**Najari et al. (** 29 **)**	2016	R	34	Microsurgical	379.4 ±164.7	515.3 ± 231.6
**Ji et al. (** 30 **)**	2016	O	260	Microsurgical	310 ± 179	669 ± 180
**Saylam et al. (** 31 **)**	2020	R	202	Microsurgical	255 ± 66	372 ± 134

O = Observational; R = Retrospective.

## DISCUSSION

Herein, the evidence that the surgical correction of varicocele improves the TT levels is seen in many studies and is corroborated by recent meta-analysis published. As we can observe, an increase of around 106 ng/dL could be achieved by the microsurgical technique (33, 34).

Furthermore, varicocele seems to cause pantesticular injury that leads to an impairment of both Sertoli and Leydig cell function, resulting in hypogonadism and altered seminal parameters and spermatogenesis. Whereas adequate intragonadal TT levels are necessary to ensure spermatogenesis (35). For example, Sirvent et al. analyzed testis biopsies from 31 men with varicocele. In addition to the atrophy of seminiferous tubules, they observed multiple changes in the characteristics of the Leydig cell population. Curiously, men with varicocele had Leydig cell hyperplasia. Sirvent et al. went further by functionally testing the expanded population of Leydig cells with the peroxidase-antiperoxidase method and demonstrated a decreased number of cells expressing testosterone. It is interesting that all men had normal peripheral levels of LH and testosterone, leading to conclude that men with varicoceles must compensate via Leydig cell hyperplasia in order to remain eugonadal (36).

Also, some trials demonstrated the improvement in seminal parameters such as motility, sperm concentration and Total Motile Sperm Count (TMSC) (20, 31) after correction of varicocele. The benefit related to improvement of FSH and LH levels remains uncertain (18, 19, 21, 31). On the other side, when the erectile function was analyzed, data show that IIEF was significantly improved by the correction of varicocele in infertile men. Consequently, these results must be interpreted cautiously given the complex interplay between infertility and sexual dysfunction (37, 38).

## CONCLUSION

Varicocele is associated with an important impairment in men's testicular function, a decreased testosterone production and a significantly increased risk for hypogonadism. Infertile men presenting with hypogonadism may benefit substantially from varicocelectomy in terms of postoperative improvements in hormonal and seminal parameters. Nevertheless, the modest rise of TT levels may not manifest clinically, and patients must be counseled not to have high expectations.

## References

[1] Meacham RB, Townsend RR, Rademacher D, Drose JA (1994). The incidence of varicoceles in the general population when evaluated by physical examination, gray scale sonography and color Doppler sonography. J Urol.

[2] Gorelick JI, Goldstein M (1993). Loss of fertility in men with varicocele. Fertil Steril.

[3] Xiao-Bin G, Fang-Lei W, Hui X, Cheng Y, Zhi-Xuan C, Zhi-Peng H (2021). The association between body mass index and varicocele: A meta-analysis. Int Braz J Urol.

[4] Kass EJ, Belman AB (1987). Reversal of testicular growth failure by varicocele ligation. J Urol.

[5] Jeremias JT, Belardin LB, Okada FK, Antoniassi MP, Fraietta R, Bertolla RP (2021). Oxidative origin of sperm DNA fragmentation in the adult varicocele. Int Braz J Urol.

[6] Valeri A, Mianné D, Merouze F, Bujan L, Altobelli A, Masson J (1993). Etude de la température scrotale chez 258 hommes sains, sélectionnés par tirage au sort dans une population d'hommes de 18 à 23 ans. Analyse statistique, observations épidémiologiques et mesure des diamètres testiculaires [Scrotal temperature in 258 healthy men, randomly selected from a population of men aged 18 to 23 years old. Statistical analysis, epidemiologic observations, and measurement of the testicular diameters]. Prog Urol.

[7] Mostafa T, Anis TH, El-Nashar A, Imam H, Othman IA (2001). Varicocelectomy reduces reactive oxygen species levels and increases antioxidant activity of seminal plasma from infertile men with varicocele. Int J Androl.

[8] Camoglio FS, Zampieri N, Corroppolo M, Chironi C, Dipaola G, Giacomello L (2004). Varicocele and retrograde adrenal metabolites flow. An experimental study on rats. Urol Int.

[9] Weiss DB, Rodriguez-Rigau LJ, Smith KD, Steinberger E (1978). Leydig cell function in oligospermic men with varicocele. J Urol.

[10] Della Morte E, Fortuna FF, Gerevini G, Lania C, Grasso M (2002). Valutazione di FSH e funzione leydigiana nel varicocele [Evaluation of FSH and Leydig cells function in patients with varicocele]. Arch Ital Urol Androl.

[11] Raboch J, Stárka L (1971). Hormonal testicular activity in men with a varicocele. Fertil Steril.

[12] Comhaire F, Vermeulen A (1975). Plasma testosterone in patients with varicocele and sexual inadequacy. J Clin Endocrinol Metab.

[13] Goldstein M, Gilbert BR, Dicker AP, Dwosh J, Gnecco C (1992). Microsurgical inguinal varicocelectomy with delivery of the testis: an artery and lymphatic sparing technique. J Urol.

[14] Lewis SEM, Esteves SC (2021). What does a varicocele do to a man's fertility? There is much more than meets the eye. Int Braz J Urol.

[15] Rodriguez-Rigau LJ, Weiss DB, Zukerman Z, Grotjan HE, Smith KD, Steinberger E (1978). A possible mechanism for the detrimental effect of varicocele on testicular function in man. Fertil Steril.

[16] Su LM, Goldstein M, Schlegel PN (1995). The effect of varicocelectomy on serum testosterone levels in infertile men with varicoceles. J Urol.

[17] Cayan S, Kadioglu A, Orhan I, Kandirali E, Tefekli A, Tellaloglu S (1999). The effect of microsurgical varicocelectomy on serum follicle stimulating hormone, testosterone and free testosterone levels in infertile men with varicocele. BJU Int.

[18] Pierik FH, Abdesselam SA, Vreeburg JT, Dohle GR, De Jong FH, Weber RF (2001). Increased serum inhibin B levels after varicocele treatment. Clin Endocrinol (Oxf).

[19] Di Bisceglie C, Bertagna A, Baldi M, Lanfranco F, Tagliabue M, Gazzera C (2007). Varicocele sclerotherapy improves serum inhibin B levels and seminal parameters. Int J Androl.

[20] Ozden C, Ozdal OL, Bulut S, Guzel O, Koyuncu HH, Memis A (2008). Effect of varicocelectomy on serum inhibin B levels in infertile patients with varicocele. Scand J Urol Nephrol.

[21] Rodriguez Peña M, Alescio L, Russell A, Lourenco da Cunha J, Alzu G, Bardoneschi E (2009). Predictors of improved seminal parameters and fertility after varicocele repair in young adults. Andrologia.

[22] Gat Y, Gornish M, Belenky A, Bachar GN (2004). Elevation of serum testosterone and free testosterone after embolization of the internal spermatic vein for the treatment of varicocele in infertile men. Hum Reprod.

[23] Lee RK, Li PS, Goldstein M (2007). Simultaneous vasectomy and varicocelectomy: indications and technique. Urology.

[24] Zohdy W, Ghazi S, Arafa M (2011). Impact of varicocelectomy on gonadal and erectile functions in men with hypogonadism and infertility. J Sex Med.

[25] Hsiao W, Rosoff JS, Pale JR, Greenwood EA, Goldstein M (2011). Older age is associated with similar improvements in semen parameters and testosterone after subinguinal microsurgical varicocelectomy. J Urol.

[26] Tanrikut C, Goldstein M, Rosoff JS, Lee RK, Nelson CJ, Mulhall JP (2011). Varicocele as a risk factor for androgen deficiency and effect of repair. BJU Int.

[27] Sathya Srini V, Belur Veerachari S (2011). Does varicocelectomy improve gonadal function in men with hypogonadism and infertility? Analysis of a prospective study. Int J Endocrinol.

[28] Abdel-Meguid TA, Farsi HM, Al-Sayyad A, Tayib A, Mosli HA, Halawani AH (2014). Effects of varicocele on serum testosterone and changes of testosterone after varicocelectomy: a prospective controlled study. Urology.

[29] Najari BB, Introna L, Paduch DA (2017). Improvements in Patient-reported Sexual Function After Microsurgical Varicocelectomy. Urology.

[30] Ji B, Jin XB (2017). Varicocele is associated with hypogonadism and impaired erectile function: a prospective comparative study. Andrologia.

[31] Saylam B, Çayan S, Akbay E (2020). Effect of microsurgical varicocele repair on sexual functions and testosterone in hypogonadal infertile men with varicocele. Aging Male.

[32] Bernie HL, Goldstein M (2018). Varicocele Repair Versus Testosterone Therapy for Older Hypogonadal Men with Clinical Varicocele and Low Testosterone. Eur Urol Focus.

[33] Klaver M, Dekker MJHJ, de Mutsert R, Twisk JWR, den Heijer M (2017). Cross-sex hormone therapy in transgender persons affects total body weight, body fat and lean body mass: a meta-analysis. Andrologia.

[34] Esposito A, Vigone MC, Polizzi M, Wasniewska MG, Cassio A, Mussa A (2022). Effect of initial levothyroxine dose on neurodevelopmental and growth outcomes in children with congenital hypothyroidism. Front Endocrinol (Lausanne).

[35] Hayden RP, Tanrikut C (2016). Testosterone and Varicocele. Urol Clin North Am.

[36] Sirvent JJ, Bernat R, Navarro MA, Rodriguez Tolra J, Guspi R, Bosch R (1990). Leydig cell in idiopathic varicocele. Eur Urol.

[37] Smith JF, Walsh TJ, Shindel AW, Turek PJ, Wing H, Pasch L (2009). Sexual, marital, and social impact of a man's perceived infertility diagnosis. J Sex Med.

[38] Wischmann TH (2010). Sexual disorders in infertile couples. J Sex Med.

